# Trends in antipsychotic prescribing for approved and unapproved indications to Medicaid-enrolled youth in Philadelphia, Pennsylvania between 2014 and 2018

**DOI:** 10.1186/s12888-021-03533-3

**Published:** 2021-10-22

**Authors:** Molly Candon, Siyuan Shen, Oluwatoyin Fadeyibi, Joseph L Smith, Aileen Rothbard

**Affiliations:** 1 Penn Center for Mental Health, Department of Psychiatry, Perelman School of Medicine, University of Pennsylvania, 3535 Market Street, 3rd Floor, Philadelphia, PA 19104 USA; 2 Leonard Davis Institute of Health Economics, University of Pennsylvania, Philadelphia, PA USA; 3 Community Behavioral Health, Philadelphia, PA USA; 4 HealthCore, Inc., Wilmington, DE USA; 5 School of Social Policy and Practice, University of Pennsylvania, Philadelphia, PA USA

**Keywords:** Antipsychotic prescribing, Medicaid, Off-label prescribing, Prior authorization, Foster care

## Abstract

**Background:**

Antipsychotic prescribing to Medicaid-enrolled youth has been the target of numerous policy initiatives, including prior authorization and quality monitoring programs, which often target specific populations. Whether these efforts have changed the level or composition of antipsychotic prescribing is unclear.

**Methods:**

Using 2014–2018 administrative claims data for Medicaid enrollees aged 21 years and under in Philadelphia, Pennsylvania,

we measured antipsychotic prescription fills overall and for youth without an approved indication (autism, bipolar disorder, or psychosis). We then assessed whether trends differed for populations that have been targeted by policy initiatives, including younger children and foster care-enrolled youth. We also identified the most common approved and unapproved indications and examined whether the treatment duration of antipsychotic prescriptions differed based on whether the youth had an approved or unapproved indication.

**Results:**

Overall, the number of Medicaid youth with an antipsychotic prescription fill halved between 2014 and 2018. Youth aged 17 years and under and foster care-enrolled youth, who were targeted by prior authorization and quality improvement efforts, experienced larger declines. Roughly half of prescriptions were for unapproved indications in both 2014 and 2018; the most common unapproved indication was ADHD, and the treatment duration was shorter for unapproved indications compared to approved indications.

**Conclusions:**

Antipsychotic prescribing to Medicaid-enrolled youth is declining, particularly among populations that have been targeted by policy initiatives like prior authorization and quality monitoring programs. Despite the fact that these initiatives often assess diagnostic criteria, half of antipsychotic prescriptions were for unapproved indications in both 2014 and 2018. More research is needed to gauge whether this prescribing is appropriate.

## Introduction

Beginning in the 1980s, antipsychotics were increasingly prescribed to children and adolescents in the United States (U.S.) [1, 2]. The uptick in antipsychotic prescribing, particularly among vulnerable populations like Medicaid enrollees and youth in foster care, raised concerns about the appropriateness of prescribing given the risks involved, including weight gain and side effects like tardive dyskinesia [3–8].

To date, the Food and Drug Administration (FDA) has approved antipsychotic use for bipolar disorder, psychosis, and symptoms associated with autism, and there were additional concerns that antipsychotics were being prescribed excessively to children with unapproved indications, such as attention deficit/hyperactivity disorder (ADHD) [9–12]. Approval from regulatory agencies, including the FDA, indicate that there is enough rigorous scientific support demonstrating that the clinical benefits of antipsychotic use for a given population and diagnosis outweigh the risk of adverse events [13].

However, prescribing without an FDA-approved indication is an important element of clinical practice, and studies have shown it is safe and efficacious when there is scientific support for its use [14]. Yet off-label prescribing—which encompasses prescribing for unapproved indications, as well as unapproved age categories, dosages, or method of administration—frequently occurs with little to no scientific support, especially in psychiatry [13, 15]. This may increase the risk of adverse drug events and improper medication management [10, 16, 17].

There is some evidence that antipsychotic prescribing is declining, but studies are inconsistent. A claims-based study found that the number of publicly-insured youth in the U.S. receiving antipsychotic prescriptions fell nearly 40% from 2008 to 2013 [18]. A more recent claims-based study also found a decline in antipsychotic prescribing from 2009 through 2017, but the study was restricted to privately-insured youth aged 2 to 7 in the U.S. [19] In contrast, a national survey in the U.S. reported increases in antipsychotic prescribing through 2014 [20]. Another national survey in the U.S. reported decreases in antipsychotic prescribing among toddlers, a plateau among elementary school-age children, and an increase among adolescents between 1999 and 2014 [21].

The inconsistent trends may be attributable to the policy environment, which includes various initiatives geared toward antipsychotic prescribing [22]. For example, thirty-one state Medicaid programs in the U.S. implemented prior authorization for antipsychotic prescribing to Medicaid-enrolled youth by 2015, which requires pre-approval from the insurer or managed care organization prior to a prescription fill [22]. Fifteen states also incorporated clinical review and other quality monitoring programs by 2015 [23]. Some of these initiatives targeted specific populations, e.g., Pennsylvania launched a quality monitoring program focused exclusively on antipsychotic prescribing to foster care-enrolled youth [24, 25].

Studies have found that approaches like prior authorization effectively decrease prescribing rates, including antipsychotic prescribing [26–28]. But it is unclear whether these efforts have had disproportionate effects on certain populations, such as foster care-enrolled youth. It is also unclear whether they are more likely to reduce prescribing to youth without approved indications, although a recently published study in Texas between 2013 and 2016, which examined unapproved antipsychotic use among publicly-insured children and adolescents, found a reduction in the proportion of antipsychotic prescriptions without an approved indication [29]. Diagnostic criteria are often included in prior authorization forms and in quality monitoring programs, which could change the composition of antipsychotic prescribing [ 30].

Using administrative claims data for a large cohort of Medicaid-enrolled youth living in Philadelphia, Pennsylvania between 2014 and 2018, this study provides a relatively up-to-date assessment of antipsychotic prescribing. We separately estimate trends in antipsychotic prescribing for approved versus unapproved indications, then examine whether trends are more pronounced in certain populations, including foster care-enrolled youth.

## Methods

### Study setting and population

Our study sample consisted of Medicaid enrollees aged 0 through 21 years in Philadelphia County who filled at least one antipsychotic prescription between January 1, 2014 and December 31, 2018. Philadelphia, Pennsylvania is the sixth largest city in the U.S., with a population of 1.6 million [31]. Philadelphia is among the poorest large cities in the U.S., and roughly a quarter of its population lives below the poverty line [32]. The racial/ethnic distribution is 8% Asian, 44% Black, 15% Hispanic, and 45% white [32].

Medicaid is the publicly-funded insurer of millions of Americans, primarily low-income adults, youth, and individuals with disabilities; in Philadelphia, over 650,000 individuals were enrolled in Medicaid in 2018 [32]. Medicaid is the primary insurer for youth enrolled in foster care, sometimes called out-of-home care, which refers to children and adolescents under 18 years old who have been temporarily removed from their familial home and placed with either relatives or unrelated foster parents [33].

### Statistical analyses

We used administrative claims data to measure antipsychotic prescription fills which could be identified using National Drug Codes outlined by the Healthcare Effectiveness Data and Information Set (HEDIS) quality measure that focuses on antipsychotic prescribing [34]. Following other claims-based studies, we defined approved indications based on whether the youth had at least one approved diagnostic code (i.e., autism spectrum disorder, bipolar disorder, and/or psychosis) in the same calendar year as a given antipsychotic prescription fill [35, 36]. Diagnoses were identified using outpatient behavioral health claims and International Classification of Disease codes (ICD-9 in 2014 and ICD-10 afterwards). Sociodemographic characteristics of youth were drawn from the Medicaid eligibility file.

We first documented antipsychotic prescribing trends over time, overall and by gender and race/ethnicity, and separately measured trends among children and adolescents who did not have an approved indication. We then examined the potential impact of two initiatives in Philadelphia—the rollout of prior authorization for antipsychotic prescribing and a quality monitoring program focused on foster care-enrolled youth, which was commissioned by the Pennsylvania Office of Mental Health and Substance Abuse [24, 28]—by separately estimating trends for youth aged 17 years and younger, who were targeted by prior authorization, and for youth enrolled in foster care, whose prescriptions were under increased scrutiny due to the quality monitoring program. Differences in the number of Medicaid-enrolled youth with an antipsychotic prescription between 2014 and 2018 (overall, by age category, foster care status, gender, and race/ethnicity) were assessed using two-tailed tests at the 95% level of significance.

Given our interest in unapproved indications, we identified the top approved and unapproved diagnoses associated with an antipsychotic prescription fill in 2018. We also compared the treatment duration, as measured by days supplied (30 days and under, 31–60 days, 61–90 days, 91–180 days, and 181–365 days), of antipsychotic prescriptions for approved versus unapproved indications in 2018.

The study was approved by the Institutional Review Board of the City of Philadelphia and the University of Pennsylvania.

## Results

**Fig. 1:**
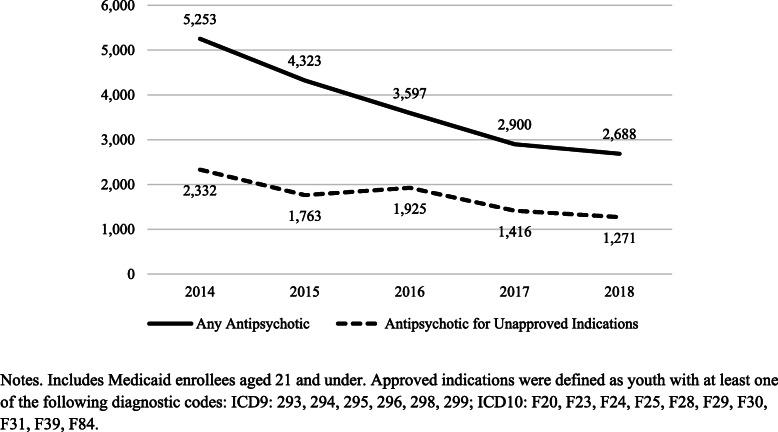
Number of Medicaid-Enrolled Youth in Philadelphia, PA who Filled Antipsychotic Prescriptions, 2014–2018

Between 2014 and 2018 there was a 49% decline (*p* < 0.001) in the number of youths with an antipsychotic prescription fill—from 5253 to 2688 (Fig. 1). When adjusting for fluctuations in Medicaid enrollment, the rate of antipsychotic prescribing fell from 17 per 1000 Medicaid-enrolled youth in 2014 to 8 per 1000 Medicaid-enrolled youth in 2018 (*p* < 0.001).

The characteristics of youth receiving antipsychotic prescriptions changed significantly as well (Table 1). Between 2014 and 2018, the average age increased from 13.8 years to 15.2 years (*p* < 0.001) and the proportion of females increased by over four percentage points (*p* < 0.001).

**Table 1:** Characteristics of Youth Enrolled in Medicaid in Philadelphia, PA Filling Antipsychotic Prescriptions, 2014 versus 2018

	Any Antipsychotic Rx			Antipsychotic Rx without an Approved Indication		
	*2014*	*2018*	*p-value*	*2014*	*2018*	*p-value*
Number of Youth	5254	2688	< 0.001	2332	1271	< 0.001
Male	64.0%	59.5%	< 0.001	70.2%	54.9%	< 0.001
Age						
< 5 years^a^	1.0%	0.4%	0.002	1.6%	0.4%	0.001
5–10 years^a^	24.5%	16.4%	< 0.001	32.2%	18.4%	< 0.001
11–17 years^a^	50.5%	44.5%	< 0.001	52.1%	49.2%	0.098
18–21 years	24.0%	38.7%	< 0.001	14.1%	32.0%	< 0.001
Race/Ethnicity						
Black	59.6%	60.6%	0.006	52.4%	58.5%	< 0.001
Hispanic	14.8%	16.4%	0.004	10.8%	14.2%	0.003
White	22.0%	16.1%	< 0.001	34.3%	23.4%	0.007
other	3.6%	6.8%	< 0.001	2.5%	4.1%	< 0.001
Foster care^b^	36.8%	30.4%	< 0.001	35.1%	35.0%	0.948

Notes. Differences between 2014 and 2018 were assessed using t-tests. Approved indications were defined as youth with at least one of the following diagnostic codes: ICD9: 293, 294, 295, 296, 298, 299; ICD10: F20, F23, F24, F25, F28, F29, F30, F31, F39, F84

^a^Youth aged 17 and under were subject to prior authorization

^b^Foster care-enrolled youth were subject to additional monitoring through a statewide antipsychotic dashboard

Notably, the reduction in antipsychotic prescribing was more pronounced among Medicaid enrollees aged 17 and under (particularly children aged 10 and under) and foster care-enrolled youth, two groups that were targeted by local prescribing initiatives. By comparison, the decrease was more modest among Medicaid enrollees between the ages of 18 and 21 years, who were not targeted in prior authorization efforts and had aged out of the foster care system.

While the raw number of antipsychotic prescriptions for unapproved indications fell between 2014 and 2018, the share of antipsychotic prescribing for unapproved indications increased slightly: 44.4% in 2014 versus 47.3% in 2018. Females (*p* < 0.001) and Black (*p* < 0.001) and Hispanic (*p* = 0.003) youth comprised a substantially larger share of prescriptions for unapproved indications in 2018 compared to 2014.

The most common FDA-approved diagnosis among youth with an antipsychotic prescription fill in 2018 was autism, followed by bipolar disorder and psychosis. The most common unapproved diagnosis was ADHD, followed by depression, adjustment disorders (which includes post-traumatic stress disorder), and conduct disorders (Table 2).

**Table 2:** Approved and Unapproved Indications Associated with Antipsychotic Prescriptions among Medicaid-enrolled Youth, 2018

**Approved Indications**	***N*** **= 1417**
Autism	41%
Bipolar Disorder	40%
Psychosis	36%
**Unapproved Indications**	***N* = 1271**
Attention deficit/hyperactivity disorder	51%
Major depressive disorder	39%
Adjustment disorders	37%
Conduct disorders	35%
Persistent mood disorders	30%
Anxiety disorders	10%
Homicidal/suicidal ideations	10%

Notes. Includes Medicaid enrollees aged 21 years and under. Diagnoses were drawn from behavioral health claims and occurred in the same calendar year as the antipsychotic prescription fill. If a youth had an approved indication, they were not tabulated in the unapproved indications

**Fig. 2:**
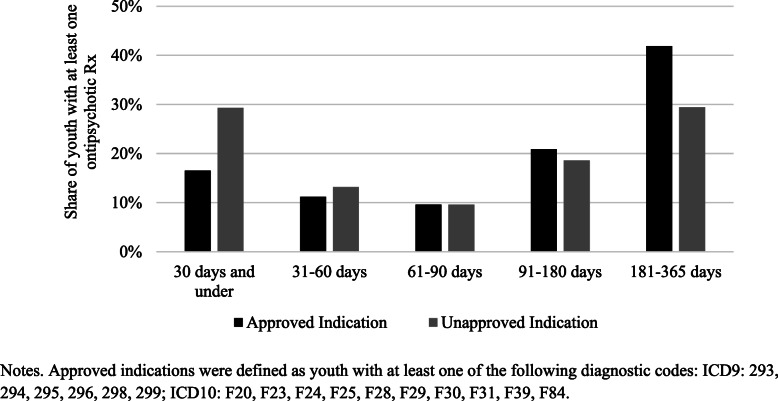
Annual Treatment Duration for Antipsychotic Prescriptions to Youth Enrolled in Philadelphia Medicaid, 2018

On average, antipsychotic prescriptions for approved indications tended to have longer treatment durations—nearly 30% of youth without an approved indication had antipsychotic prescriptions that were 30 or fewer days compared to 16% of youth with approved indications (Fig. 2).

## Discussion

This study measured trends in antipsychotic prescribing to Medicaid-enrolled youth residing in Philadelphia, Pennsylvania, finding a large reduction in antipsychotic prescribing between 2014 and 2018. The decline was concentrated among Medicaid enrollees who were 17 years of age and under, which suggests that the drop-off was associated with age requirements for prior authorization that were introduced by Pennsylvania Medicaid fee-for-service in 2006 and expanded to Medicaid managed care organizations in Philadelphia [22, 28]. The decline was also concentrated among foster care-enrolled youth, who were subject to a statewide quality monitoring program [24].

While overall rates declined, we found an increase in the proportion of antipsychotics prescribed to female patients. There could be a number of factors driving this trend, but one possibility is the historic discrepancy in diagnoses for certain conditions based on gender is narrowing. Studies have found that females have been under-diagnosed for approved and unapproved indications commonly associated with antipsychotic use, such as autism and ADHD, due to the lack of recognition and referral bias [37, 38]. More awareness of clinical symptoms in female patients could increase the likelihood that they receive a psychiatric diagnosis and accompanying antipsychotic prescription.

Both prior authorization and quality monitoring for foster care-enrolled youth assessed diagnostic criteria for antipsychotic prescribing, yet we found that the share of antipsychotic prescriptions for unapproved indications remained steady—nearly half of prescriptions occurred for unapproved indications in both 2014 and 2018. While few studies have assessed the impact of these types of initiatives on the composition of prescribing, this finding contrasts a recent study of youth in Texas Medicaid [29]. Our finding also contrasts a study of a prior authorization program that focused exclusively on diagnostic criteria for gabapentin prescriptions, which reported a significant decline in gabapentin prescribing for unapproved indications [39].

Providers have cited many reasons for relying on off-label prescribing to patients in psychiatry, including the dearth of effective medications for mood swings and aggression [40, 41]. When we explored the diagnoses of youth with antipsychotic prescription fills in 2018, we found high rates of ADHD, conduct disorders, and other conditions that are sometimes marked by these behavioral symptoms.

One dimension of antipsychotic prescribing that has not been explored, to our knowledge, is whether there are differences in the duration of antipsychotic use for approved and unapproved indications. We found that prescriptions for unapproved indications were shorter on average—antipsychotic prescriptions that lasted 30 or fewer days comprised nearly 30% of prescriptions for unapproved indications, nearly double the rate of prescriptions for approved indications. This suggests that providers are taking a more cautious approach when prescribing to youth without an approved indication.

While these findings may not be generalizable to other populations, studies have found that youth enrolled in Medicaid are over three times more likely to receive an antipsychotic prescription than youth with commercial insurance, making this an important population to study [42]. We face other limitations. Following other claims-based studies, our approach to determining whether antipsychotic prescriptions were for approved indications was based on available diagnoses in Medicaid behavioral health claims in a given year [28, 29]. Without chart review, it is unclear whether providers were targeting key symptoms that warranted antipsychotic prescribing. For example, if providers were targeting symptoms associated with autism spectrum disorder but did not specify autism spectrum disorder in insurance claims, the antipsychotic prescription would be categorized as unapproved [43]. Another limitation is the study period, which begins in 2014 and ends in 2018 (data standardization and availability prevent us from analyzing more recent years of data and the full impact of policies like prior authorizations). Given these limitations, we cannot conclusively estimate the causal effects of policy initiatives.

## Conclusion

Despite these limitations, our findings have important implications. Antipsychotic prescribing to Medicaid-enrolled youth in Philadelphia is declining dramatically. However, the share of antipsychotic prescribing for unapproved indications has been relatively steady and females and Black and Hispanic youth comprised a larger share of youth with antipsychotic prescriptions for unapproved indications in 2018 compared to 2014, which may be driven by disparities in quality or access to health care. More research is needed to understand whether these trends reflect more judicious antipsychotic prescribing or if the drop-off in antipsychotic prescribing has resulted in unintended effects, particularly among those without affordable and accessible treatment alternatives.

## Data Availability

Our primary data source are Medicaid claims, which are not publicly accessible. The SAS code used to generate the findings is available upon request. Requests can be sent to the corresponding author at molly.candon@pennmedicine.upenn.edu, 3535 Market Street, 3rd Floor, Philadelphia, PA 19104, U.S.

## Abbreviations

FDA: Food and drug administration
ICD: International classification of disease
ADHD: Attention deficit/hyperactivity disorder
